# Dual Approach to Depression: The Combined Efficacy of Intermittent Hypoxia and Fluoxetine in Modulating Behavioral and Inflammatory Responses

**DOI:** 10.3390/biomedicines12092116

**Published:** 2024-09-18

**Authors:** Francini Arboit, Gabriele Cheiran Pereira, Maria Fernanda Pessano Fialho, Gabriela Becker, Evelyne da Silva Brum, Micheli Mainardi Pillat, Guilherme Vargas Bochi, Luiz Osório Cruz Portela, Eliane Maria Zanchet

**Affiliations:** 1 Center of Health Sciences, Graduate Program in Pharmacology, Federal University of Santa Maria, Santa Maria 97105-900, Brazil; fraarboit@hotmail.com (F.A.); gabrielecheiran@gmail.com (G.C.P.); mmpillat@gmail.com (M.M.P.); guilherme.bochi@ufsm.br (G.V.B.); 2 Center of Natural and Exact Sciences, Graduate Program in Biological Sciences: Biochemistry Toxicology, Federal University of Santa Maria, Santa Maria 97105-900, Brazil; mariafpessano@outlook.com (M.F.P.F.); becker.gabi@hotmail.com (G.B.); esbrum.eb@gmail.com (E.d.S.B.); 3 Center of Health Sciences, Department of Physiology and Pharmacology, Federal University of Santa Maria, Santa Maria 97105-900, Brazil; 4 Center of Physical Education and Sports, Federal University of Santa Maria, Santa Maria 97105-900, Brazil; luizzportel@gmail.com

**Keywords:** stress, major depression, interleukins, intermittent hypoxia

## Abstract

**Background/Objectives:** Mental disorders pose a significant public health challenge, affecting millions worldwide. Given the limitations of current therapies, many patients experience inadequate responses and adverse effects. Intermittent hypoxia (IH) has demonstrated anxiolytic, antidepressant, and neuroprotective properties in various protocols. This study investigated the effects of acute IH (13% O_2_, 1 h), fluoxetine (FLX) and their combination on depression-like behavior, serum corticosterone, and inflammatory cytokine levels induced by acute restraint stress in C57BL/6 female mice. **Methods:** Behavioral assessments included the tail suspension test, forced swim test, and open field test. **Results:** The combined IH + FLX treatment exhibited a synergistic effect, reducing immobility time and increasing latency time, respectively, in the tail suspension test (46%, *p* = 0.0014; 73%, *p* = 0.0033) and forced swim test (56%, *p* = 0.0082; 48%, *p* = 0.0322) compared to the ARS group. Biochemical analysis revealed that individual and combined treatments significantly reduced most inflammatory interleukins by up to 96%. Corticosterone levels were reduced by 30% only in the IH group. **Conclusions:** These findings highlight the potential of a one-hour IH session, particularly when combined with fluoxetine, to alleviate depressive-like behaviors and exert anti-inflammatory effects, suggesting a promising therapeutic approach for depression.

## 1. Introduction

Mental health disorders, which significantly impact both the physical and psychological well-being of individuals, pose a substantial public health concern, diminishing overall quality of life. Major Depressive Disorder (MDD), the most prevalent among neuropsychiatric disorders, affects approximately 5% of adults globally [1], highlighting the widespread impact of depression on public health, with a higher incidence rate observed in pregnant women, the elderly, children, and other vulnerable populations [2]. MDD is characterized by persistent negative mood, diminished interest in daily activities, fatigue, feelings of guilt and worthlessness, sleep disturbances, and often suicidal ideation [2,3,4].

MDD is a multifaceted and heterogeneous disorder with a pathophysiology that remains unclear. However, numerous lines of evidence suggest that MDD stems from a combination of genetic, psychological, and environmental factors [2]. Stress, in particular, emerges as a crucial element associated with the development of this pathology [5,6,7,8,9], increasing the individual susceptibility. Acute restraint stress (ARS) is often used as an experimental model of depression in animals [10,11,12]. Beyond inducing depression and anxiety-like behaviors in animals, this model causes alterations in physiological systems, leading to elevated pro-inflammatory cytokines, increased serum corticosterone levels, and oxidative stress in brain regions implicated in these disorders [13,14,15,16].

Pharmacological treatment of MDD with antidepressants aims to manage the symptoms [17], but the lack of consistently effective approaches is particularly problematic. Resistance and risk of relapse progressively increase with successive treatment courses [18]. This challenge is compounded by the fact that approximately 30% of patients exhibit no clinical improvement or only a partial response to treatment, which often necessitates weeks to months to achieve full therapeutic effects, accompanied by considerable side effects [19,20,21]. Consequently, the limitations of current pharmacotherapies underscore an urgent need for improved treatment options for individuals with MDD. Developing novel approaches encompassing both pharmacological and non-pharmacological interventions is key to addressing this important challenge in today’s neurobiology and medicine.

Currently, antidepressant drugs are considered the primary and most effective pharmacotherapy for depression [22]. Selective serotonin reuptake inhibitors (SSRIs), such as fluoxetine, are among the most commonly prescribed pharmaceutical drugs for the treatment of depression and other mood disorders [23].

Non-pharmacological intervention, such as intermittent hypoxia, has been suggested as a beneficial tool to prevent depression and as a therapy, including those cases of refractory depression [24]. IH is the exposure to repeated or recurrent episodes of low oxygen content (hypoxia), interspersed with periods of normoxia (21% O_2_). It is currently used for the acclimatization of pilots and mountaineers, increasing the performance of athletes, and for the treatment/prevention of diseases such as hypertension, ischemic coronary artery disease, Parkinson’s disease, and acute myeloid leukemia [16,25,26]. Other studies demonstrate that IH has anxiolytic, antidepressant, and neuroprotective effects [27,28]. Furthermore, IH has been shown to be effective in preventing and treating damage induced by post-traumatic stress models [29,30].

Considering the significant public health impact of Major Depressive Disorder (MDD) and the shortcomings of traditional treatments, our hypothesis is that the combination of intermittent hypoxia (IH) and fluoxetine (FLX) may provide synergistic therapeutic benefits in alleviating depression-like behaviors and associated biochemical alterations.

## 2. Materials and Methods

### 2.1. Animals

Experiments in our study followed the National Research Council’s Guide for the Care and Use of Laboratory Animals. Our Institutional Animal Care and Use Committee approved all experimental protocols (Process number 23081.020892/2020-60). C57BL/6 female mice (20–25 g, 60–90 days old) were obtained from the Central Animal Facility of the Federal University of Santa Maria and housed under standard conditions: 22 ± 2 °C temperature, a 12 h light–dark cycle, and ad libitum access to food and water. Animals were acclimated for seven days before the experiments began.

### 2.2. Drugs and Treatments

Fluoxetine (Fluoxetina, Medley^®^) was prepared in a 0.9% NaCl isotonic saline solution and administered orally (p.o.) using the gavage technique at a dosage of 10 mg/kg [31,32]. The IH sessions were conducted within an acrylic chamber designed to ensure proper animal containment. The air composition was rapidly and precisely adjusted using a gas compressor GO2 Altitude Hypocator (Biomedtech Australia Pty Ltd., Melbourne, Australia). For the current experiment, an oxygen (O_2_) concentration of 13% was selected. The IH program comprised 15 min of hypoxic exposure followed by 5 min of reoxygenation, totaling 1 h per session. The specifics of this protocol were determined based on prior research conducted by our group [33].

### 2.3. Acute Restraint Stress (ARS) Procedure

This study used the acute restraint stress (ARS) model of animal depression. Animals were confined in modified 50 mL Falcon tubes with air circulation holes for 6 h, following the established protocol [34]. Cotton disks were used to adjust tube length, restricting head and limb movement to ensure normal breathing without causing pain.

### 2.4. Experimental Design

**Figure 1:**
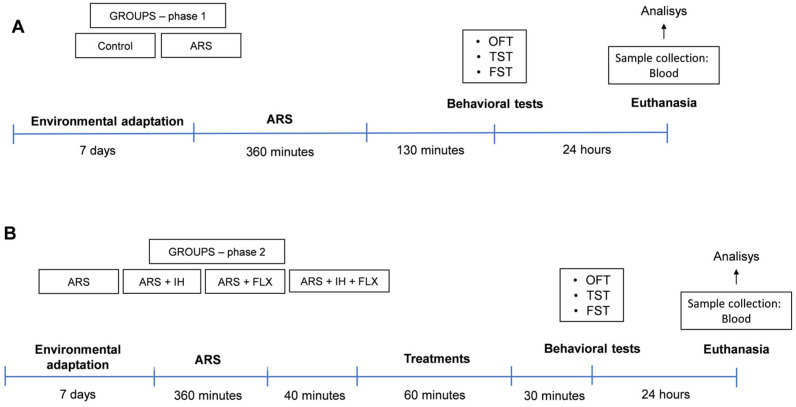
Schematic overview of the experimental design. (**A**) Phase 1: Depressive-like behavior induction by the ARS protocol to investigate behavioral and biochemical changes in mice. (**B**) Phase 2: Evaluation of treatment effects on behavioral and biochemical parameters in animals subjected to the ARS protocol. CONTROL: mice that did not receive any treatment; ARS: mice subjected to six hours of restraint; ARS + IH: mice exposed to six hours of restraint and treated with intermittent hypoxia (13% O_2_, 1 h); ARS + FLX: mice exposed to six hours of restraint and treated with fluoxetine (10 mg/kg, p.o.); ARS + FLX + IH: mice exposed to six hours of restraint and treated with fluoxetine (10 mg/kg, p.o.) in combination with intermittent hypoxia (13% O_2_, 1 h); OFT: open field test; TST: tail suspension test; FST: forced swim test; *n* = 6 mice/group.

The study was conducted in two phases during the light phase of the light–dark cycle. To avoid behavioral interference, all tests were recorded on video. Phase 1 involved dividing mice into two groups (*n* = 6 animals/group): a control group and an ARS group exposed to a 6 h protocol. Behavioral tests (OFT, TST, FST) were conducted 130 min post-ARS. Twenty-four hours later, animals were euthanized, and blood was collected for biochemical analysis (see Figure 1A for the experiment timeline). In Phase 2 (Figure 1B), subsequent to Phase 1 observations, the focus shifted to treatment effects. Initially, all animals underwent the same ARS protocol and were then returned to their cages for 40 min. They were then divided into four groups (*n* = 6 animals/group): ARS, ARS + IH (13% O_2_, 1 h), ARS + FLX (10 mg/kg, p.o), or ARS + IH + FLX. Thirty minutes after treatment administration, animals underwent the same behavioral tests as in Phase 1 (OFT, TST, FST). Twenty-four hours later, animals were anesthetized and euthanized by cervical dislocation, and blood was collected for biochemical analysis.

### 2.5. Behavioral Tests

#### 2.5.1. Open Field Test (OFT)

The OFT, initially outlined by Prut and Belzung (2003) [35], evaluates locomotive and exploratory behaviors in animals. Each animal was placed individually in a 60 × 60 × 60 cm circular apparatus divided into quadrants (EP 154 Campo Aberto Open Field Acrílico Ø 60cm, INSIGHT equipamentos pesquisa-ensino, São Paulo, Brazil). Over 5 min, their activities, such as time spent in the center, total crossings, and rearings, were observed and recorded [36,37,38].

#### 2.5.2. Forced Swim Test (FST)

The FST, introduced by Porsolt and colleagues (1978) [39], assesses depressive-like behavior in animals. Each animal underwent a 6-min swim in transparent cylinder (EP 180 Nado Forçado–Ratos e Camundongos, INSIGHT equipamentos pesquisa-ensino, São Paulo, Brazil) filled with 40 cm of water at 25 ± 2 °C. Immobility, defined as passive floating, indicating demotivation, was recorded during the session. Latency to first exhibit immobility was also measured. Results were quantified by immobility and latency, both in seconds (s) [11,38,40].

#### 2.5.3. Tail Suspension Test (TST)

The TST, adapted from Steru and colleagues (1985) [41], was used in this study. It involved securing the animal’s tail with adhesive tape to a vertical surface 60 cm above the ground for 6 min. The test measured the time until the first immobility episode and the total immobility duration in seconds (s).

### 2.6. Biochemical Analysis

#### 2.6.1. Serum Corticosterone Levels

The corticosterone concentration in serum was measured using an Enzyme-Linked Immunosorbent Assay (ELISA) kit from Enzo Life Sciences (Farmingdale, NY, USA), following the manufacturer’s protocol. Results were interpolated from a standard curve and normalized logarithmically, and are reported in picograms per milliliter (pg/mL).

#### 2.6.2. Inflammatory Parameters

For inflammatory parameters, serum levels of IL-2, IL-4, IL-6, IL-17, IFN-γ, and TNF-α were assessed using flow cytometry with the BD Cytometric Bead Array Mouse Th1/Th2/Th17 Cytokine Kit (BD Biosciences, Franklin Lakes, NJ, USA). The procedures were carried out according to the manufacturer’s instructions. Serum samples were mixed with capture beads and detection reagents, incubated, and analyzed on a BD FACSCalibur flow cytometer (BD Biosciences, USA). FlowJo software version 10.8.1 (Tree Star, Ashland, OR, USA) was used for data analysis, and the results were quantified in pg/mL to determine cytokine concentrations in the serum.

### 2.7. Statistical Analysis

The data were summarized as mean ± SEM and analyzed using GraphPad Prism software (version 8.0, San Diego, CA, USA). Prior to analysis, normality was assessed using the Kolmogorov–Smirnov test. In Phase 1, differences between the control and ARS groups were evaluated using Student’s *t*-test or the Mann–Whitney test based on data distribution characteristics. In Phase 2, one-way ANOVA with Dunnett’s post hoc test or the Kruskal–Wallis test with Dunn’s post hoc test was used, depending on data distribution. Statistical significance was defined as *p* < 0.05, with the results meeting this criterion considered statistically significant.

## 3. Results

### 3.1. Phase 1

The impact of the ARS protocol on depressive-like behavior and associated biochemical changes was evaluated using Student’s *t*-test and is demonstrated in Table 1. The TST showed that, compared to the control, the ARS group had significantly increased immobility time [t(10) = 2.24, *p* = 0.04)], while latency time remained unchanged. In the FST, the ARS group exhibited lower latency [t(10) = 2.37, *p* = 0.04)] and higher immobility [t(10) = 3.76, *p* = 0.003)] compared to the control.

The OFT revealed significant differences between the groups, with the ARS group displaying fewer crossings [t(10) = 2.25, *p* = 0.04] and rearings [t(10) = 3.90, *p* = 0.002].

Furthermore, serum corticosterone levels after the ARS protocol were assessed. The Student’s *t*-test results demonstrated that the ARS group had significantly elevated values compared to the control group [t(8) = 3.71, *p* = 0.0059].

**Figure 2:**
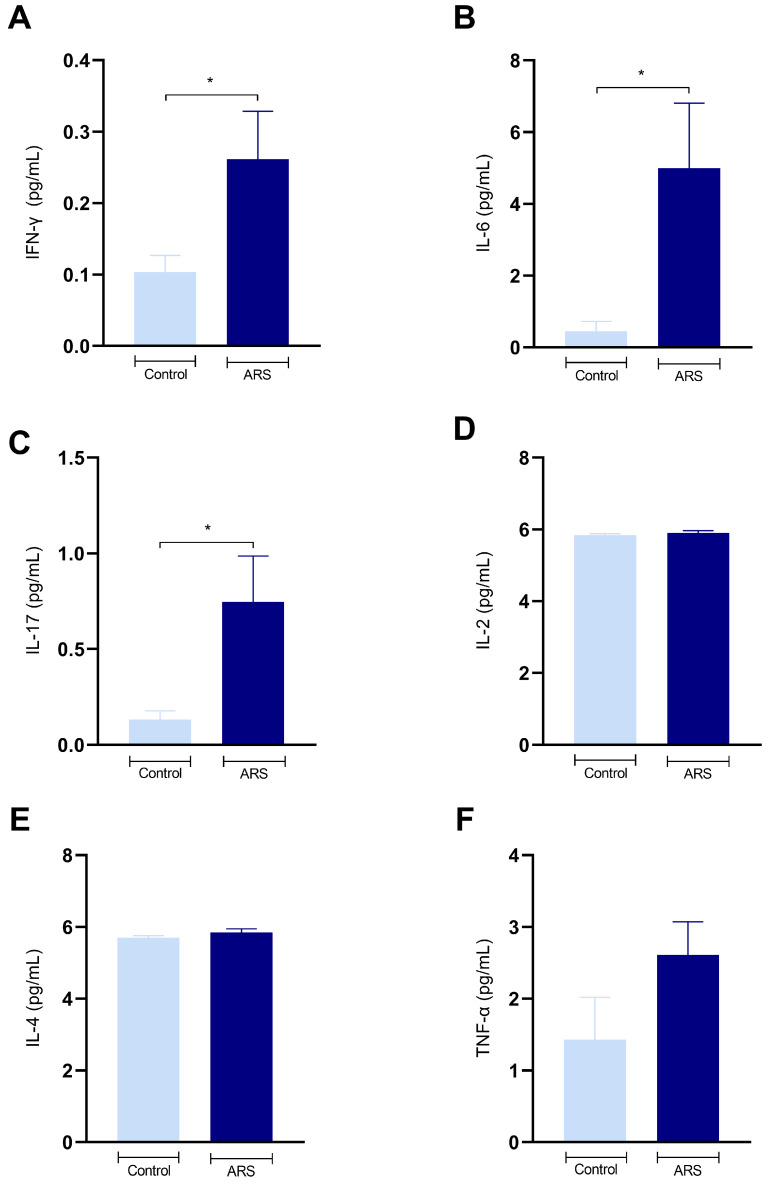
Effects of ARS on serum cytokine levels. (**A**) IFN-γ (pg/mL); (**B**) IL-6 (pg/mL); (**C**) IL-17 (pg/mL); (**D**) IL-2 (pg/mL); (**E**) IL-4 (pg/mL); (**F**) TNF-α (pg/mL). The data were obtained using Student’s *t*-test (parametric statistic) or the Mann–Whitney test (non-parametric statistics). The differences between groups are shown with asterisk symbols above the indicative bars. Values are expressed as the mean ± SEM (*n* = 6 mice/group). Significant differences: * *p* < 0.05.

To explore the potential involvement of the inflammatory process in stress-induced alterations, serum cytokine levels were evaluated. The outcomes revealed increased levels of IFN-γ [t(10) = 2.23, *p* = 0.04] (Figure 2A), IL-6 [t(10) = 2.48, *p* = 0.03] (Figure 2B), and IL-17 [t(10) = 2.29, *p* = 0.04] (Figure 2C) after the ARS protocol. However, IL-2, IL-4, and TNF-α levels did not show significant differences between the groups.

### 3.2. Phase 2

**Figure 3:**
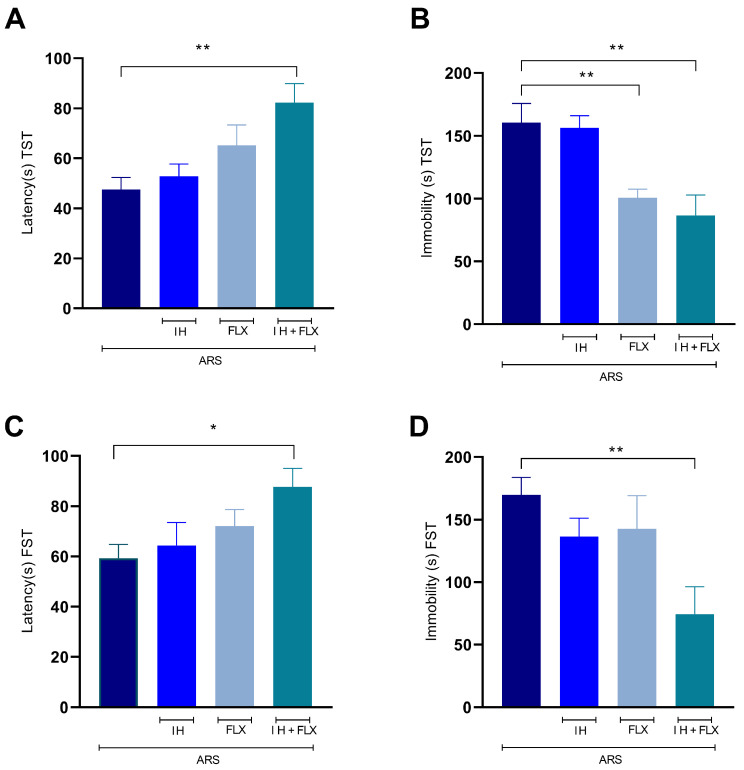
Effects of treatments on TST and FST. (**A**,**B**) tail suspension test; (**C**,**D**) forced swim test. The data were obtained using one-way ANOVA followed by Dunnet post hoc test (parametric statistics) or Kruskal–Wallis test followed by Dunn’s post hoc test (non-parametric statistics). The differences between groups are shown with asterisk symbols above the indicative bars. Values are expressed as the mean ± SEM (*n* = 6 mice/group). Significant differences: * *p* < 0.05, ** *p* < 0.01.

Utilizing one-way ANOVA for statistical analysis, the effects of treatments on both behavior and biochemical changes in the animals were assessed. In the TST, the combined treatment resulted in increased latency when compared to the ARS group [F (3,20) = 5.59, *p* = 0.0033] (Figure 3A). Additionally, the immobility time in this test (Figure 3B) was lower in the FLX- [F(3,20) = 9.02, *p* = 0.008] and the IH + FLX [F(3,20) = 9.02, *p* = 0.001]-treated groups in comparison to the stressed group. Within the FST, the IH + FLX treatment led to an increase in latency time [F(3,20) = 2.88, *p* = 0.03] (Figure 3C) and a reduction in immobility time [F(3,20) = 4.06, *p* = 0.008] (Figure 3D) compared to the ARS group.

**Figure 4:**
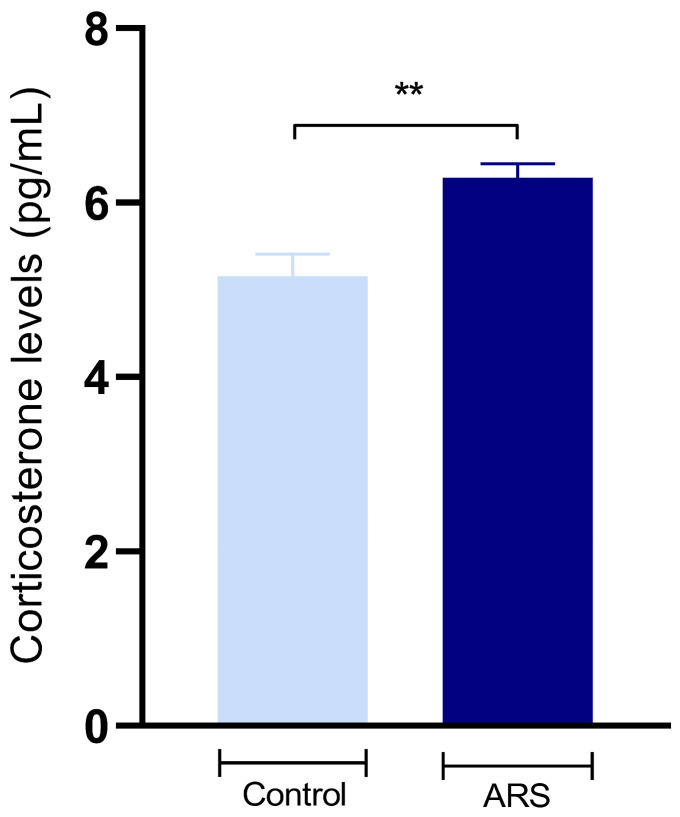
Effects of treatments on serum corticosterone levels (pg/mL). The data were obtained using one-way ANOVA followed by Dunnet post hoc test. Differences between groups are shown with asterisk symbols above the indicative bars. Values are expressed as the mean ± SEM (*n* = 5 mice/group). ** *p* < 0.01.

Furthermore, the treatments yielded discernible effects on the biochemical analyses. Notably, IH treatment significantly reduced the level of serum corticosterone in comparison to the ARS group [F(3,16) = 3.55, *p* = 0.014] (Figure 4).

**Figure 5:**
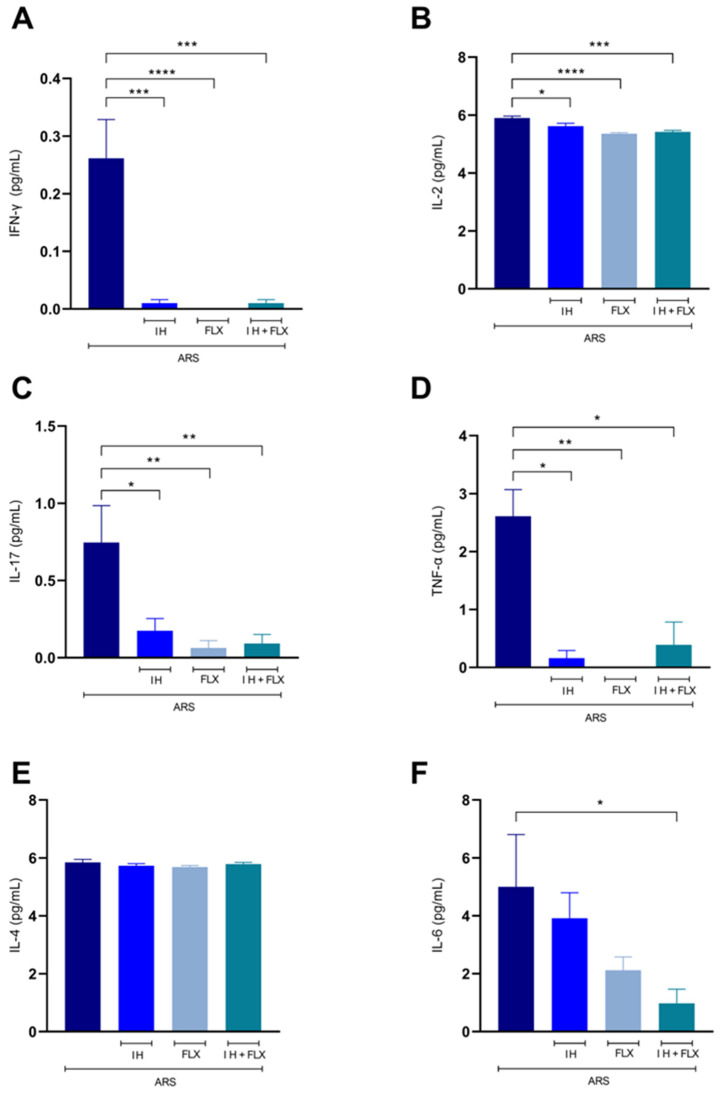
Effects of treatments on serum cytokine levels. (**A**) IFN-γ (pg/mL); (**B**) IL-2 (pg/mL); (**C**) IL-17 (pg/mL); (**D**) TNF-α (pg/mL); (**E**) IL-4 (pg/mL); (**F**) IL-6 (pg/mL). The data were obtained using one-way ANOVA followed by Dunnet post hoc test (parametric statistics) or Kruskal–Wallis test followed by Dunn’s post hoc test (non-parametric statistics). The differences between groups are shown with asterisk symbols above the indicative bars. Values are expressed as the mean ± SEM (*n* = 5 mice/group). Significant differences: * *p* < 0.05, ** *p* < 0.01, *** *p* < 0.001; **** *p* < 0.0001.

The effect of the treatments on interleukin levels was also tested. IH, FLX, and IH + FLX significantly reduced the levels of IFN-γ (Figure 5A) compared to the ARS group [F(3,20) = 14.27, *p* = 0.0001], [F(3,20) = 14.27, *p* < 0.0001], [F(3,20) = 14.27, *p* = 0.0001], respectively. These treatments also significantly decreased the levels of IL-2 (Figure 5B) [F(3,20) = 12.81, *p* = 0.02], [F(3,20) = 12.81, *p* < 0.0001], [F(3,20) = 12.81, *p* = 0.0002], respectively, as well as IL-17 (Figure 5C) [F(3,20) = 5.95, *p* = 0.01], [F(3,20) = 5.95, *p* = 0.0043], [F(3,20) = 5.95, *p* = 0.0060], and TNF-α (Figure 5D) (*p* = 0.02), (*p* = 0.0016), (*p* = 0.011). Regarding IL-6 (Figure 5F), only the combination treatment (IH + FLX) showed significant results [F(3,20) = 2.88, *p* = 0.03].

**Figure 6:**
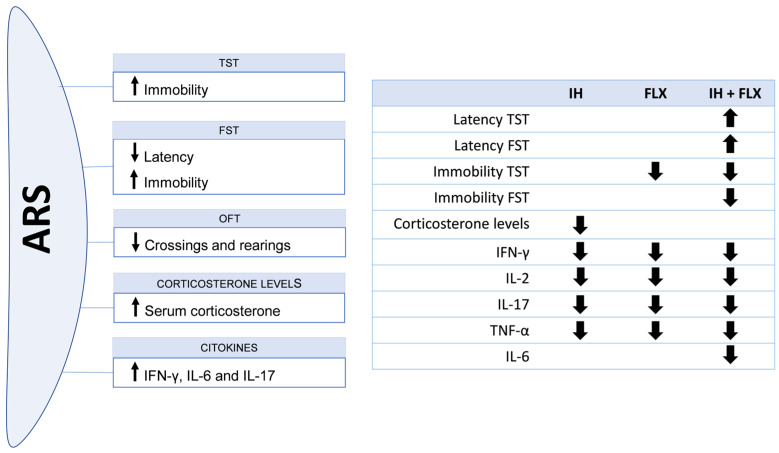
Summary of the main findings of the study. (**left**) the effects of the ARS protocol on behavioral and biochemical parameters are represented. (**right**) the effects of the treatments on the same parameters are demonstrated. Upward arrows indicate an increase, and downward arrows indicate a decrease.

The findings of this study indicate that the 6 h acute restraint stress model effectively induced depression-like behavior in mice, as evidenced by increased immobility in both the tail suspension test and forced swim test, accompanied by elevated levels of pro-inflammatory interleukins and corticosterone. The combined treatment of fluoxetine and intermittent hypoxia demonstrated significant efficacy in reversing these behaviors, marked by an increase in latency to the first immobility episode and a reduction in the overall immobility duration. Furthermore, this treatment combination led to a notable decrease in inflammatory interleukin levels. A comprehensive summary of these results is presented in Figure 6.

## 4. Discussion

In this study, we explored the potential therapeutic benefits of combining intermittent hypoxia (IH) with fluoxetine to alleviate depression-like behaviors and biochemical changes induced by acute restraint stress (ARS) in female mice. Depression is a complex disorder, often accompanied by dysregulation of mood, stress response, and inflammation. Although current treatments are effective for most patients, there is a gap that could be further studied and addressed to improve treatment efficacy, especially for non-responsive patients.

In the first stage of our study, we observed that 6 h of restraint induced behavioral alterations consistent with depressive-like behavior and changes in interleukin and corticosterone levels. The ARS group showed increased immobility in the TST and FST and reduced latency in the FST, indicating quicker onset and longer duration of immobility.

In the literature, increased immobility in these tests is associated with a measure of depressive-like behavior and behavioral despair [42]. Similar findings were reported with increased immobility and corticosterone levels after 7 h [43] and 6 h [44] of restraint. On the other hand, Misztak and colleagues [45], using the chronic restraint model, observed an increase in immobility in the TST after only three hours of restraint, but not after six. Additionally, we conducted the OFT. Following the ARS protocol, animals showed altered exploratory behavior, indicated by decreased total distance traveled and rearings, which is associated with anxiety [46,47]. This supports our TST and FST findings, emphasizing the complex relationship between anxiety and depression, which often co-occur and share similar symptoms and mechanisms [8,48].

The acute restraint stress (ARS) model also altered IFN-γ, IL-6, and IL-17 levels, results that are consistent with findings from other studies using both acute [49,50] and chronic [51] restraint models. These studies demonstrate that the physical stress induced by restraint can trigger a significant immune response, increasing the production of pro-inflammatory cytokines regardless of the duration of exposure to the stressor.

In the study’s second phase, we evaluated the effects of treatments individually and in combination. The combined approach showed an antidepressant-like effect in behavioral tests like the TST and FST, along with changes in biochemical markers linked to MDD. This study represents the first investigation into the antidepressant effects of combining IH with fluoxetine, suggesting a potential synergistic interaction between these treatments.

Synergy, as a concept, proposes that different mechanisms of action can amplify each other’s efficacy [52]. For instance, the observed synergy between fluoxetine, a selective serotonin reuptake inhibitor (SSRI) [53], and IH presents promising possibilities for depression treatment. There are no publications on the association of intermittent hypoxia with other antidepressants. However, Misztak and colleagues observed that the co-administration of fluoxetine and zinc enhanced the antidepressant-like response in the TST, suggesting a synergistic effect that could potentially improve treatment efficacy in depression models [45].

Fluoxetine’s antidepressant effects involve serotonin modulation, impacting mood, emotions, and sleep regulation [54]. Recent findings challenge the serotonin hypothesis of depression, suggesting that long-term antidepressant use may reduce serotonin levels [55]. Their mechanism of action extends beyond serotonin modulation, also impacting the glutamatergic system and increasing levels of brain-derived neurotrophic factor (BDNF), which plays a key role in neuroplasticity and neuronal survival [56,57,58].

In turn, IH triggers protective mechanisms like anti-inflammatory responses, activates antioxidants, promotes neurogenesis, increases BDNF levels, modulates neuropeptide Y, and enhances synaptic plasticity, which is crucial for tissue integrity and cell survival during stress and injury [26,59,60,61,62,63,64]. Despite its mechanical paradox, IH’s protective intensity depends on factors such as timing, severity, and duration of hypoxic challenge [65]. IH influences psychiatric disorders through mechanisms like hypoxia-inducible factor 1 (HIF-1), regulating gene expression in response to low oxygen and impacting erythropoiesis and energy metabolism [28]. In low oxygen conditions, HIF-1 adjusts cell metabolism by reducing mitochondrial reactive oxygen species and promoting glycolysis [66], which are crucial for countering elevated reactive oxygen species (ROS) levels implicated in neurological and psychiatric disorders [28]. This blend of fluoxetine and intermittent hypoxia’s varied effects and shared mechanisms might explain the success of the proposed association observed in our study. Thus, the combination of fluoxetine and IH holds promise for alleviating depression symptoms and promoting long-term brain health. However, when used in isolation, only fluoxetine reduced immobility in the TST. Other behavioral tests did not yield significant results for either isolated treatment, possibly due to variations in hypoxia protocols and the specificities of the experimental setup. The inconsistency of fluoxetine’s antidepressant effect in single-dose administration may explain the varied outcomes in different studies. While some research found no impact [31], others observed a reduction in immobility in behavioral tests [67,68].

It is known that in stressful situations, the body’s equilibrium is disturbed, impacting several physiological functions. Stress triggers the release of glucocorticoids, like cortisol in humans and corticosterone in rodents [69,70,71]. A recent systematic review pointed out that cortisol is a predictor of MDD [72]. Our study found that a single six-hour session of restraint stress increased serum corticosterone levels, indicating hyperactivity of the HPA axis in response to stress. Reversal of serum corticosterone levels through antidepressant therapy stabilizes the HPA axis in stressed mice [43]. Contrary to common beliefs about hypoxia heightening HPA axis reactivity [73,74], our findings, using a mild protocol, showed that it actually lowered corticosterone levels. The difference may be due to the severity of the hypoxia protocol, as gentler protocols can lead to beneficial adaptations [63]. This aligns with observations of other depression treatments in animal models [75,76].

Cytokines affect mood regulation and may contribute to depression, which is supported by elevated immune abnormalities in depressed individuals and the onset of depression after cytokine treatments [77,78]. Research confirms higher levels of interleukin-6 and C-reactive protein in depressed patients [79]. Our study confirms the link between stress and inflammation, as the ARS protocol impacted levels of IFN-γ, IL-17, and IL-6. Similar results were found using 6 h restraint for 28 days [51] and the LPS depression model [80]. Additionally, chronic mild stress and other depression models have shown increased levels of pro-inflammatory cytokines like IL-1β, IL-6, TNF-α, and IL-17 in both animals and humans [79,81,82,83,84].

Regarding the action of treatments on interleukins, our study demonstrated a significant reduction in IL-6 levels with the FLX + IH combination. IL-6 is one of the most consistent biomarkers of depression, with elevations observed in circulating levels [85], and is associated with treatment resistance [86]. Similar results to ours in reducing IL-6 were demonstrated in a study using a 14.3% hypoxia protocol 4 h per day for 14 or 28 days [87]. Regarding other cytokines also involved in MDD, such as IFN-γ, IL-2, TNF α, and IL-17, all treatments effectively reversed their levels. Similar results were obtained by Li and colleagues (2022) [88], who showed that intermittent hypoxic conditioning (13% O_2_) suppresses proinflammatory cytokines such as TNF-α, IL-1β, and IL-6 while upregulating anti-inflammatory cytokines like IL-10 in the hippocampus of C57BL/6 mice. These results confirm the anti-inflammatory action of IH in the CNS.

Our study investigated the relationship between interleukins and depression in females, an area often overlooked despite evidence suggesting females may have heightened inflammatory reactivity and sensitivity to social cues compared to males [89,90]. Understanding this relationship is crucial; given females’ increased susceptibility to depression [91], they are more sensitive to social cues [89] and exhibit greater inflammatory reactivity [90]. Our study found that treatments reversed interleukin increases from acute stress in females, offering promise for addressing inflammation-induced depression despite historical gender research biases. While stress models like the EPM and FST were primarily developed using male subjects and may not fully capture female-specific responses due to factors such as the estrous cycle [92,93,94], our findings indicate minimal sex-based differences in immediate stress markers during acute restraint sessions, affirming the relevance of our results across genders in short-term stress scenarios.

A limitation of our study is the lack of BDNF level measurements, a key factor given that both fluoxetine and intermittent hypoxia are known to influence BDNF signaling. Previous unpublished data from our laboratory have shown increased BDNF levels following intermittent hypoxia, underscoring its importance in neurogenesis. Additionally, the study’s acute design, both in terms of the model used and the duration of treatment, presents another limitation, as depression treatments are typically chronic. Although the results presented with just one hour of treatment are promising, as they already demonstrated effects, the outcome with chronic treatment could be even more significant. This short-term approach limits our ability to fully assess the long-term therapeutic potential of the interventions. Our next step is to study the effects of chronic treatment (IH + FLX) in a chronic restraint stress model, which may offer more insight into the mechanisms over time.

Our study highlights the potential of combining intermittent hypoxia with fluoxetine as a new treatment for depression. IH, already used in humans for athletic performance and cardiovascular health, may also be useful in psychiatry. By modulating inflammation, promoting neurogenesis, and affecting neurotransmitters, IH could complement current antidepressants. This opens the door for further exploration, especially in cases where conventional treatments are less effective or patients show resistance.

## 5. Conclusions

The results of this study involving one hour of intermittent hypoxia combined with fluoxetine are promising. This single treatment session showed significant improvements in behavioral tests and stress-related biomarkers. The combined treatment had a synergistic effect, reducing immobility time and increasing latency time in the TST and FST, which was not observed with individual treatments. Biochemical analysis indicated that both individual and combined treatments significantly reduced levels of most inflammatory interleukins. These findings suggest that a one-hour IH session, especially when combined with fluoxetine, has the potential to alleviate depressive-like behaviors and exert anti-inflammatory effects. This innovative approach holds promise as a potential therapeutic option for depression. However, further research is essential to assess its long-term effectiveness and underlying mechanisms. Only through additional studies, particularly those investigating chronic therapy and confirming the safety and efficacy of this combination, can this method be regarded as a viable therapeutic option.

## Figures and Tables

**Table 1:** ARS-induced behavioral changes.

Parameters	Control	ARS	*p*-Value
Latency TST	55.17 ± 3.32	47.50 ± 4.84	0.2209
Immobility TST	119.2 ± 10.28	160.5 ± 15.27	0.0485
Latency FST	87.33 ± 11.28	59.33 ± 5.40	0.0493
Immobility FST	93.67 ± 14.54	169.7 ± 14.03	0.0037
Number of crossings	90.33 ± 3.83	79.67 ± 3.29	0.0474
Number of rearings	31.33 ± 2.41	20.50 ± 1.36	0.0029
Corticosterone levels	5.15 ± 0.25	6.28 ± 0.16	0.0059

Note: values are expressed as mean ± SEM. Abbreviations: ARS: acute restraint stress; FST: forced swim test; TST: tail suspension test.

## Data Availability

The original contributions presented in the study are included in the article; further inquiries can be directed to the corresponding author.
